# LncRNAs H19 and HULC, activated by oxidative stress, promote cell migration and invasion in cholangiocarcinoma through a ceRNA manner

**DOI:** 10.1186/s13045-016-0348-0

**Published:** 2016-11-03

**Authors:** Wen-Tao Wang, Hua Ye, Pan-Pan Wei, Bo-Wei Han, Bo He, Zhen- Hua Chen, Yue-Qin Chen

**Affiliations:** 1 Key Laboratory of Gene Engineering of the Ministry of Education, State Key Laboratory for Biocontrol, Sun Yat-sen University, Guangzhou, 510275 People’s Republic of China; 2 Department of Hepatobiliary, Sun Yat-sen Memorial Hospital, Sun Yat-sen University, Guangzhou, 510120 People’s Republic of China; 3 Department of Anesthesiology, Sun Yat-sen Memorial Hospital, Sun Yat-sen University, Guangzhou, 510120 People’s Republic of China

**Keywords:** Inflammation response, Oxidative stress, ceRNA, Cholangiocarcinoma, Migration and invasion

## Abstract

**Background:**

Long non-coding RNAs (lncRNAs) are known to play important roles in different cell contexts, including cancers. However, little is known about lncRNAs in cholangiocarcinoma (CCA), a cholangiocyte malignancy with poor prognosis, associated with chronic inflammation and damage to the biliary epithelium. The aim of the study is to identify if any lncRNA might associate with inflammation or oxidative stress in CCA and regulate the disease progression.

**Methods:**

In this study, RNA-seqs datasets were used to identify aberrantly expressed lncRNAs. Small interfering RNA and overexpressed plasmids were used to modulate the expression of lncRNAs, and luciferase target assay RNA immunoprecipitation (RIP) was performed to explore the mechanism of miRNA-lncRNA sponging.

**Results:**

We firstly analyzed five available RNA-seqs datasets to investigate aberrantly expressed lncRNAs which might associate with inflammation or oxidative stress. We identified that two lncRNAs, H19 and HULC, were differentially expressed among all the samples under the treatment of hypoxic or inflammatory factors, and they were shown to be stimulated by short-term oxidative stress responses to H_2_O_2_ and glucose oxidase in CCA cell lines. Further studies revealed that these two lncRNAs promoted cholangiocyte migration and invasion via the inflammation pathway. H19 and HULC functioned as competing endogenous RNAs (ceRNAs) by sponging let-7a/let-7b and miR-372/miR-373, respectively, which activate pivotal inflammation cytokine IL-6 and chemokine receptor CXCR4.

**Conclusions:**

Our study revealed that H19 and HULC, up-regulated by oxidative stress, regulate CCA cell migration and invasion by targeting IL-6 and CXCR4 via ceRNA patterns of sponging let-7a/let-7b and miR-372/miR-373, respectively. The results suggest that these lncRNAs might be the chief culprits of CCA pathogenesis and progression. The study provides new insight into the mechanism linking lncRNA function with CCA and may serve as novel targets for the development of new countermeasures of CCA.

**Electronic supplementary material:**

The online version of this article (doi:10.1186/s13045-016-0348-0) contains supplementary material, which is available to authorized users.

## Background

CCA is broadly described as malignancies originating from a malignant transformation of cholangiocytes and epithelial cells lining the intra-hepatic and extra-hepatic biliary ducts. CCA is difficult to diagnose and cure, partly due to the unknown molecular mechanisms underlying the development of CCA. In the past two decades, several studies have described molecular details regarding the malignant transformation of cholangiocytes and the environment of chronic inflammation in bile ducts with subsequent cholangiocyte damage, which is most commonly believed to contribute to CCA pathogenesis [1, 2]. A variety of inflammatory cytokines, including IL-6, IL-8, and TGFβ [2, 3], were found to contribute to the apoptosis, senescence, migration, invasion, and cell cycle regulation of malignant cholangiocytes [4]. Oxidative stress, potentially caused by *Clonorchis sinensis*, *Opisthorchis viverrini*, hepatitis B virus (HBV) infection, or inflammation [5–8], is also implicated in CCA development. The increased reactive oxygen species (ROS) produced by oxidative stress can damage lipids, proteins, and DNA and subsequently alter different cellular pathways and influence gene expression, which may induce tumor promotion and progression [9]. Persistent oxidative stress might also induce inflammatory responses, implying a complex association between oxidative stress and inflammation in CCA [7]. Previous studies have reported that malignant transformation of cholangiocytes was associated with chronic inflammation in the biliary epithelium; however, detailed molecular mechanisms of CCA promotion and progression are still unclear.

Recently, promising evidence has shown that non-coding RNAs serve as ideal diagnostic biomarkers and participate in oxidative stress and disease or cancer-related inflammation [10–14]. Small non-coding RNAs, especially microRNAs (miRNAs), are widely reported to play vital roles in disease pathogenesis, including the pathogenesis of CCA, and their post-transcriptional regulatory mechanisms [15, 16]. Several miRNAs, such as miR-370 [17], miR-373 [18, 19], and miR-21 [20, 21], are dysregulated and play vital roles in CCA. However, none of these miRNAs have been indicated to regulate inflammation or oxidative stress.

In addition to miRNAs, a set of long non-coding RNAs (lncRNAs), that are more than 200 nucleotides in length, have also been reported to play pivotal roles in a variety of diseases. Some lncRNAs, including ANRIL [22], HOTAIR [23], and H19 [24], influence many processes in various cancers, including chromatin remodeling, X chromosomal inactivation, transcriptional regulation, molecular trafficking [25], inflammation responses, and oxidative stress [10–12, 14]. These observations suggest potential pathophysiological contributions from lncRNAs in CCA and imply that some lncRNAs might be involved in the inflammation response pathways stimulated by infection. Recently, the mechanisms and functions of several lncRNAs, such as lncRNA-HEIH [26], HULC [27], and HOTAIR [28], were uncovered in hepatic carcinoma, the most common hepatic malignancy, and these findings led to the construct of regulation networks and extended our knowledge regarding tumorigenesis in hepatic carcinoma. Similarly, dysregulated lncRNAs in CCA may play an important role in CCA promotion and progression through involvement in CCA key pathways, such as inflammation or oxidative stress. Currently, no lncRNAs have been reported to be associated with CCA progression.

In this study, we sought to determine if any lncRNA is stimulated by oxidative stress and trigger inflammation in CCA. We identified a set of lncRNAs that participated in inflammation and oxidative stress response. Two dysregulated lncRNAs H19 and HULC were shown to promote cell migration and invasion through ceRNA manners leading to the activation of inflammation cytokine IL-6 and chemokine receptor CXCR4. Our findings may provide potential biomarkers for CCA progression and potential therapeutic targets for the disease.

## Methods

### Cell lines

QBC939 human cholangiocarcinoma cells were obtained from Shuguang Wang (The Third Military Medical University, China). SK-cha-1 human cholangiocarcinoma cells were kindly provided by Dr. Chundong Yu (Xiamen University, China). RBE human cholangiocarcinoma cells and HEK293T human embryonic kidney cells were purchased from the Type Culture Collection of the Chinese Academy of Sciences (Shanghai, China). All CCA cell lines were cultured in RPMI-1640 medium with 10 % fetal calf serum. HEK-293 T cells were cultured in DMEM medium with 10 % fetal calf serum. All cells were maintained in a 37 °C humidified incubator with 5 % CO_2_.

### qRT-PCR analysis

Cells were collected in EP tubes. Then, total RNA was extracted from tissue or cells using TRIzol reagent (Invitrogen, Carlsbad, CA, USA) following the manufacturer’s instructions. RNA was reverse-transcribed into cDNA with the ReverTra Ace® qPCR RT Kit (TOYOBO, Japan) or PrimeScript® RT reagent Kit with gDNA Eraser (Takara, Japan). Real-time PCR for lncRNA was performed using the SYBR Premix ExTaq real-time PCR kit (Takara, Japan) according to the manufacturer’s instructions with GAPDH as normalization control. The expression level for each lncRNA and mRNA was determined using the 2^−△△Ct^ method. The specificity and reliability of the PCR were confirmed by sequencing the PCR product fragments. All primers are shown in Additional file 1: Table S1.

### Oxidative stress treatment in vitro

CCA cells were treated with 1 μM H_2_O_2_ for 1 h and 1 μM glucose oxidase for 24 h for short-term and long-term oxide incubation in vitro, respectively.

### Vector construction

Full-length H19-pcDNA 3.1 vectors were purchased from Integrated Biotech Solutions (Shanghai, China). The pcDNA 3.1 vector was used for lncRNA and mRNA overexpression, and the psiCHECK-2 vector was used for the luciferase target assay. Primers and oligonucleotides for full-length HULC, IL-6, and CXCR4 amplification and 3′-UTR segments of IL-6 and CXCR4 construction are listed in Additional file 1: Table S1.

### Cell transfection and luciferase target assay

We plated 1.6 × 10^4^ RBE cells or 5 × 10^4^ HEK-293 T cells in 48-well plates. Then, 500 ng psiCHECK-2-derived reporter vectors (Promega, Madison, WI, USA), 500 ng pcDNA3.1 vectors overexpressing lncRNAs (Invitrogen Corporation, Carlsbad, CA, USA), and 30 nM miRNA mimics (GenePharma, Shanghai, China) were co-transfected with Lipofectamine® LTX (Invitrogen Corporation, Carlsbad, CA, USA) and Lipofectamine® 3000 (Invitrogen Corporation, Carlsbad, CA, USA). Finally, 48 h after transfection, cells were harvested for the dual luciferase reporter assay (Promega, Madison, WI, USA). Each experiment was repeated at least three times.

### Protein extraction, western blotting, and antibodies

As previously described, 72 h after transfection, the RBE cells were harvested, and total protein was extracted from the cells using RIPA lysis buffer (Beyotime Biotechnology, Shanghai, China) with 1× complete ULTRA (Roche, Nutley, USA). The proteins were detected with antibodies against CXCR4 (ab2074, Abcam, USA), IL-6 (SC-7920, Stantacruz, USA), GAPDH (Proteintech Technology, Manchester, UK), and β-tubulin (32−2600, Invitrogen, USA). The protein levels were normalized to GAPDH or β-tubulin.

### RNA immunoprecipitation (RIP)

In the assay, the enrichment of miRNAs binding with AGO2, a key component of the microRNA-containing RISC complex, were used as the positive controls, and U6 as the negative. The AGO2 (FB261-S2, Abnova, Taiwan) monoclonal primary antibody was rip grade, and the procedure of rip experiment was followed to the manual of Magan rip Kit (17−700, Millipore, USA).

### Scratch wound healing assay

Transfected RBE cells were cultured in 24-well plates for 24 h in standard conditions until 80−100 % confluency was reached. Linear wound tracks were generated with sterile, 10-μl pipettes and maintained under standard conditions. The scratched cells were rinsed twice with PBS to remove non-adherent cells, and fresh RPMI-1640 medium (with no fetal calf serum) was added. Photographs of the centers of the gaps were taken using a 10× phase-contrast microscope (Zeiss). Cell migration at 0 and 24 h after scratching was evaluated by determining the wound distance at two random wound gap locations. Three independent scratch wound experiments were used for calculations.

### Migration and invasion assays

Migration and invasion assays were conducted using Transwell chambers (8 μm, Corning Costar Co., Cambridge, USA) according to the manufacturer’s instructions. For the migration assays, 8 × 10^4^ RBE cells transfected after 24 h were plated in the top chambers lined with a non-coated membrane. For the invasion assay, the chamber inserts were coated with a 1:9 deliquation of Cultrex® Basement Membrane Extract (Trevigen, USA). Then, 1.2 × 10^5^ cells were plated in the top chamber, while 600 μl RPMI-1640 with 10 % fetal bovine serum was added to the lower chamber as a chemoattractant. After a 24-h incubation at 37 °C, cells located in the lower chamber were fixed with 100 % methanol and stained with 0.1 % crystal violet. Cells were counted in ten fields for triplicate membranes at 10× magnification using a microscope (Zeiss). Five random sights in each sample were selected to analyze cell count, and the mean of triplicate experiments was calculated.

## Results

### H19 and HULC involved in inflammation-associated pathways triggered by oxidative stress in CCA cell lines

**Fig. 1:**
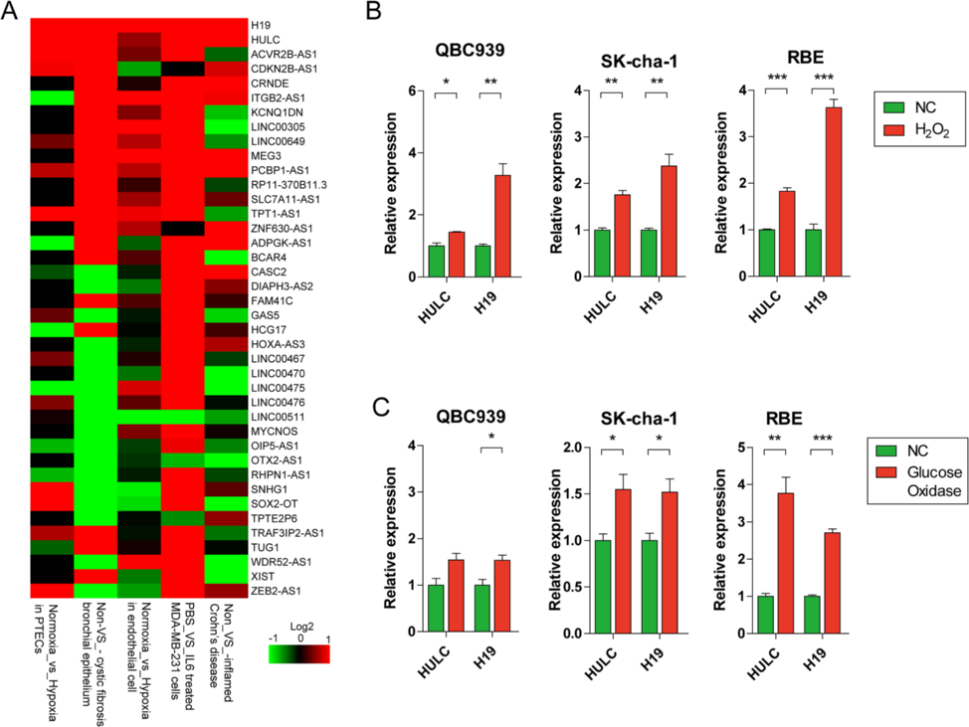
Analysis of Inflammation-associated lncRNAs. **a** Heat maps of differential lncRNA profiles are based on studies upon lncRNAs responded to hypoxic and inflammatory factor. Each of the GEO data has suggested the aberrantly lncRNA related with inflammation and/or oxidative stress. For example, GSE76743 identified endothelial cell response to hypoxia. Others: GSE57539 (PBS_VS_IL6 treated MDA-MB-231 cells), GSE67106 (Non_ VS_ inflamed Crohn’s disease), GSE55146 (Non_ VS_ cystic fibrosis bronchial epithelium), and GSE70544 (Normoxia_vs_Hypoxia in proximal tubular epithelial cells (PTECs)). **b** LncRNAs stimulated by short-term oxidative stress using H_2_O_2_ in QBC939, SK-cha-1, and RBE cells. **c** LncRNAs stimulated by long-term oxidative stress using glucose oxidase in QBC939, SK-cha-1, and RBE cells

In human bile duct cells, inflammatory responses were usually triggered by oxidative stress, which was induced by HBV, HCV, or excretory-secretory proteins released by *C. sinensis* or *O. riverine* [6, 8]. We speculated that the lncRNAs stimulated by oxidative stress may trigger inflammation in CCA. To identify whether any lncRNA related with inflammation is regulated by oxidative stress, we firstly reanalyzed several GEO data, GSE76743 [29], GSE57539 [30], GSE67106 [31], GSE55146 [32], and GSE70544 [33], and identified 40 lncRNAs responding to oxidative stress or inflammatory response (Fig. 1a). Among these, XIST, CDKN2B-AS1, CRNDE, TUG1, SOX2-OT, etc. have been reported playing crucial roles in inflammation pathway of cancer progression. Importantly, two lncRNAs, HULC and H19, showed higher expressed among all of the five selected datasets, suggesting that they may involve in inflammation pathway that is regulated by oxidative stress. To validate the expression patterns of HULC and H19 under oxidative stress, we then used three human CCA cell lines, QBC939, SK-cha-1, and RBE, for short-term or long-term oxide incubation using H_2_O_2_ and glucose as described in previous studies [34] and measured the expression levels of HULC and H19 by qPCR. The results showed that both lncRNAs were significantly up-regulated in three cell lines (Fig. 1b), suggesting a potential function in acute oxidative stress responses. Similarly, HULC and H19 were also significantly up-regulated in at least two cell lines after long-term oxidative stress induced by glucose oxidase (Fig. 1c), and these two lncRNAs might be more likely to contribute to the pathogenesis of CCA through chronic inflammation caused by long-term oxidative stress. It is worth noting that HULC and H19 were up-regulated after both short- and long-term oxidative stress, implying their pivotal roles in inflammation promotion and CCA pathogenesis. Additionally, we have also investigated the heme oxygenase-1 (HO-1) gene expression, which were reported to have the cytoprotective effect under various pathophysiological stimulating conditions, such as oxidative stress [35]. As a result, the expression of HO-1 had increased significantly under the oxidative stress condition (Additional file 1: Figure S1), suggesting that the HO-1 may also protect cholangiocarcinoma from oxidative stress [36, 37].

### lncRNAs H19 and HULC regulate cholangiocyte migration and invasion

**Fig. 2:**
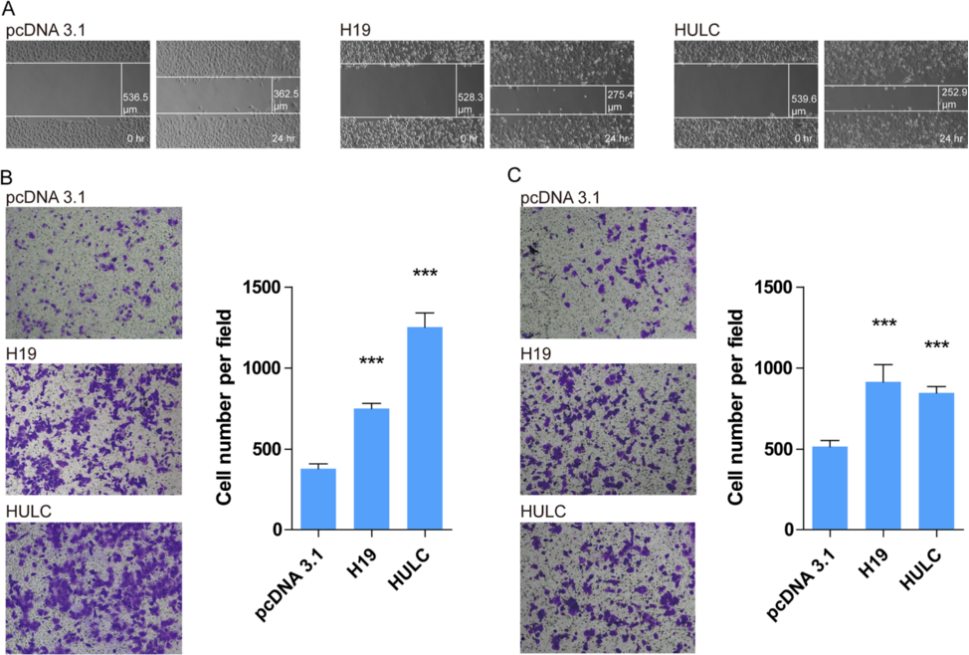
Enforced H19 and HULC expression increased the ability of migration and invasion in CCA cells. **a** Scratch wound healing assays of CCA cells. **b** Migration assays of CCA cells. **c** Invasion assays of CCA cells

Combined with previous observations, we speculated that H19 and HULC are stimulated by oxidative stress and regulate inflammation pathways involved in CCA pathogenesis. To validate this speculation, we investigated the function of H19 and HULC in CCA cells. Several inflammation pathways are involved in CCA by modifying migration and invasion; therefore, we used scratch wound healing assays to further demonstrate the function of H19 and HULC in migration potency. After 24 h in culture, CCA cells with forced expression of H19 or HULC (Additional file 1: Figure S2A) dramatically promoted wound closure compared to the control (Fig. 2a), suggesting that H19 and HULC have migration regulating roles. We also performed migration assays and invasion assays to validate the migration and invasion abilities of H19 and HULC. Consistence with the scratch wound healing assays, H19 and HULC significantly expedited migration (2.0-fold for H19 and 3.3-fold for HULC, Fig. 2b) and invasion (1.77-fold for H19 and 1.64-fold for HULC, Fig. 2c), which further strengthened the migration and invasion roles of H19 and HULC in CCA. Furthermore, we also investigated the migration and invasion abilities of RBE cells when knocking down H19 or HULC. The results showed that the migration (Additional file 1: Figure S3A) and invasion (Additional file 1: Figure S3B) abilities of RBE cells were decreased when H19 or HULC was knocked down (Additional file 1: Figure S2B). These data imply that H19 and HULC may regulate the inflammation factors regulating migration and invasion after stimulating oxidative stress.

### H19 and HULC sponge a set of miRNAs and the inflammation factors

**Fig. 3:**
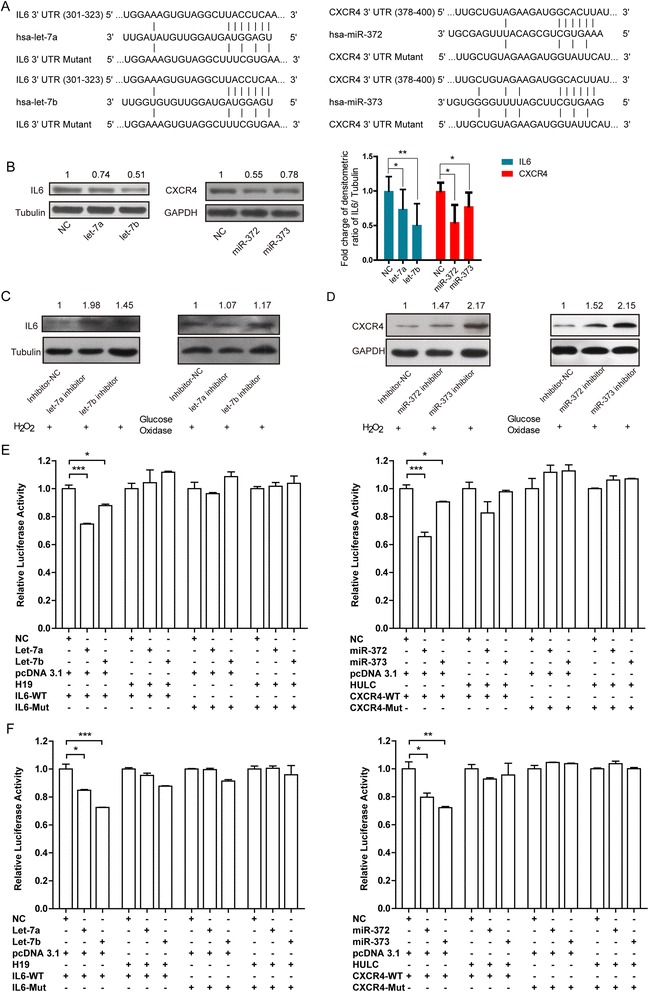
H19 and HULC regulated genes via ceRNA behaviors. **a** Schematic representation of the interaction between miRNAs and the 3'-UTR of IL-6 or CXCR4. **b** Let-7a/let-7b and miR-372/miR-373 reduced the abundance of IL-6 and CXCR4 proteins, respectively in CCA cells. The right histogram analyzed the relative fold charges of protein levels. Triplicate experiments were analyzed by mean ± SD, *p* value <0.01, **, <0.05, *. MiRNA down-regulation by transfecting the miRNA inhibitors into RBE cells under oxidative stress, and the expression levels of IL-6 (**c**) and CXCR4 (**d**) increased significantly. Enforced expressed of H19 and HULC rescued luciferase activity for vectors with the 3'-UTR of IL-6 or CXCR4, which were suppressed by let-7a/let-7b and miR-372/miR-373, respectively, in RBE cells (**e**), and in HEK-293 T cells (**f**)

We further investigated the potential target genes of H19 and HULC and the underlying regulatory mechanisms. Previous studies showed that both H19 and HULC are similar to ceRNAs [38] in human embryonic kidney cells and human embryonal carcinoma cells by sponging let-7 and miR-372, respectively [27, 39]. To determine whether H19 and HULC have similar roles in CCA, we focused on the potential functions of the let-7 and miR-372 families in CCA cells (Additional file 1: Figure S4A). We used bio-informational predicting and found the let-7 might target IL-6, one of the most important inflammatory factors in the pathogenesis, growth and migration of CCA [40], and miR-372 and miR-373 might target CXCR4, a chemokine receptor involved in CCA progression and metastasis [41] (Fig. 3a). Subsequently, a dual luciferase reporter system (Additional file 1: Figure S4B) and a western bolt assay (Fig. 3b) in RBE CCA cells had identified the direct regulation of IL-6 and CXCR4 by let-7a/let-7b and miR-372/miR-373, respectively. We also performed miRNA down-regulation experiment by transfecting the miRNA inhibitors into RBE cells under oxidative stress and found that the expression levels of IL-6 and CXCR4 increased significantly (Fig. 3c, d, Additional file 1: Figure S5). These results revealed that the activation of these mRNAs over oxidative stress is miRNA-dependent.

### lncRNAs H19 and HULC function through a ceRNA pattern in the inflammation pathway

**Fig. 4:**
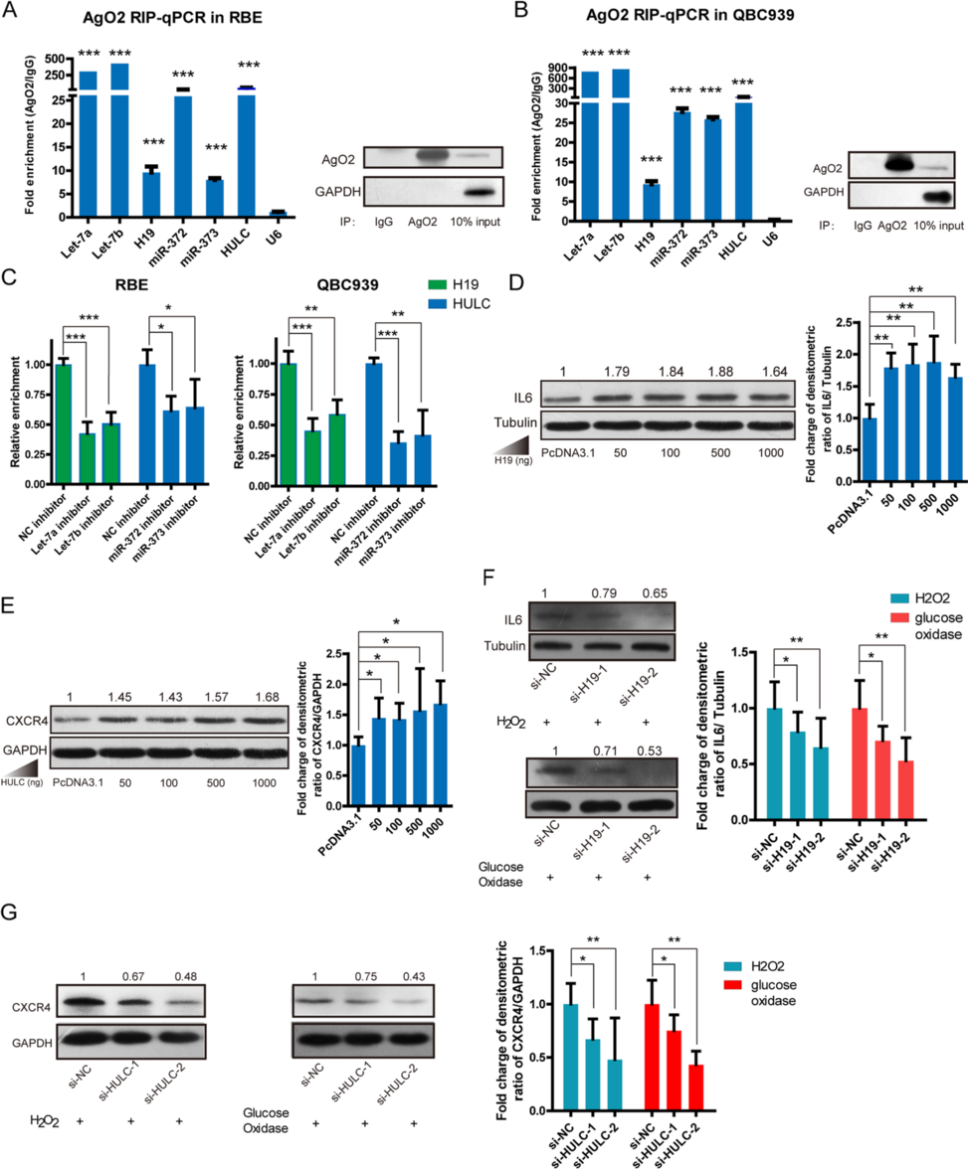
The mechanism of H19 and HULC that regulated genes. **a** AGO2 rip-qPCR of miRNAs and lncRNAs in RBE cells. The enrichment of miRNAs binding with AGO2 were used as the positive controls and U6 as the negative. **b** AGO2 rip-qPCR of miRNAs and lncRNAs in QBC939 cells. Triplicate experiments were analyzed by mean ± SD, *p* value <0.001, ***. **c** Relative enrichment of H19 (*green*) and HULC (*blue*) were analyzed in AGO2 rip-qPCR with let-7a, let-7b inhibitor or miR-372, miR-373 that compared to inhibitor NC. Enforced expression of different concentration of H19 (**d**) and HULC (**e**) plasmid increased IL-6 and CXCR4 protein levels, respectively, in RBE cells. The expression of IL-6 (**f**) and CXCR4 (**g**) had decreased significantly when we knocked down H19 and HULC, respectively, under the oxidative stress condition. The right histogram of gray density to quantitatively analyze the relative fold charges of protein levels. Triplicate experiments were analyzed by mean ± SD, *p* value < 0.001, ***, <0.01, **, <0.05, *

To further confirm whether H19 and HULC regulate inflammation and CCA pathogenesis through ceRNA patterns, we co-transfected miRNAs, 3′-UTR, and lncRNAs in HEK-293 T and RBE cells. Compared with empty vectors, we found that overexpressed lncRNAs rescued the luciferase activity suppressed by miRNAs (Fig. 3e in RBE cells and 3f in HEK-293 T cells), suggesting that H19 up-regulates IL-6 by suppressing let-7a and let-7b, and HULC up-regulates CXCR4 by suppressing miR-372 and miR-373. Moreover, we further performed a RNA immunoprecipitation (RIP) experiment to investigate the direct binding of H19, HULC to their certain miRNAs. In the assay, the enrichment of miRNAs binding with AGO2, a key component of the microRNA-containing RISC complex, were used as the positive controls and U6 as the negative [42]. The RIP results revealed that H19 and HULC were associated with the AGO2 protein in CAA cells (Fig. 4a, b). Moreover, we detected the AGO2-immunoprecipitated lncRNAs with or without miRNAs that knocked down by inhibitors. The results showed that the enrichment of AGO2-immunoprecipitated H19 and HULC was decreased in the let-7a/7b or miR-372/373 inhibitors transfected into RBE cells (Fig. 4c), indicating that H19 and HULC are directly binding to the certain miRNAs, let-7a/7b, miR-372/373, and function as ceRNA manner in CAA cells. The protein levels were also measured and overexpressed H19 or HULC in RBE cells increased the abundance of IL-6 and CXCR4 proteins, respectively (Fig. 4d, e). Additionally, we further testified that both of their target genes IL-6 and CXCR4 are down-regulated when H19 and HULC are knocked down, respectively, in RBE cells under the oxidative stress condition (Fig. 4f, g). Functional validation also indicated that forced expression of IL-6 and CXCR4 plays a similar role to H19 and HULC in promoting cell migration and invasion in CCA cells (Additional file 1: Figure S6 and S7). These results suggested that H19 and HULC regulate cell migration and invasion by increasing IL-6 and CXCR4 levels through sponging let-7a/let-7b and miR-372/miR-373, respectively.

**Fig. 5:**
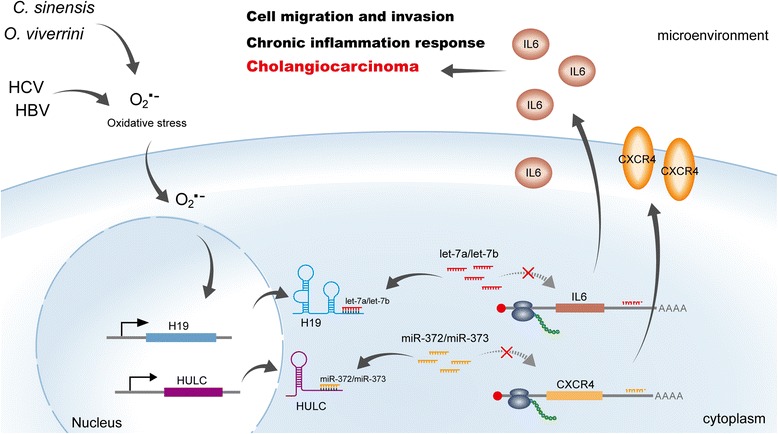
Schematic representations of pathways modulated by H19 and HULC in CCA cells. H19 and HULC are up-regulated by oxidative stress induced by viruses or liver flukes and sponging miRNAs, such as let-7a/let-7b and miR-372/miR-373. This increases the expression of inflammation-related genes, including IL-6 and CXCR4, resulting in abnormal inflammation responses and pathogenesis of CCA

In conclusion, our results suggest that two inflammation-associated lncRNAs in CCA, H19 and HULC, target inflammatory genes, including IL-6 and CXCR4, using ceRNA methods and primarily regulated cell migration and invasion (Fig. 5). These lncRNAs might serve as key regulators in CCA.

## Discussion

The importance of several dysregulated lncRNAs has been implicated in developmental regulation and disease pathogenesis, and the function of many dysregulated lncRNAs have been reported in various cancers [28, 43–45]. However, no studies on lncRNAs associated with CCA migration and invasion have been reported. In this study, we found that H19 and HULC targeted IL-6 and CXCR4*,* respectively, by sponging miRNAs in CCA cells. The study provides new insight into the mechanism linking lncRNA function with CCA and may serve as novel targets for the development of new countermeasures of CCA.

A number of studies have revealed that IL-6 was released by autocrine and paracrine by malignant cells or immune cells and functioned in tumorigenesis [40, 46, 47]. Generally, IL-6 was secreted by noncancer stem cells in low-attachment culture conditions and enriched Oct4 gene expression by activating the IL-6R/JAK/STAT3 signaling pathway in chronic inflammatory disease, such as cholangiocarcinoma [47, 48]. Our previously study also found treatment of CAA cells with IL-6 can active paracrine IL-6/STAT3 pathway in inflammation and CCA initiation [49]. IL-6 is a multifunctional inflammatory cytokine that plays a major role in the response of cholangiocytes to inflammation [40, 50]; and increased concentrations of IL-6 during inflammation in the biliary tract stimulates several pathways, including the JAK-STAT pathway, the p38 MAPK pathway, the ERK pathway, and the PI3-kinase pathway, and is involved in survival and growth of malignant cholangiocytes [40, 51]. CXCR4 is a chemokine receptor involved in several inflammatory processes and diseases, including CCA [52], and induces CCA cell migration and invasion via the ERK 1/2 and Akt pathways [53, 54]. However, a single lncRNA can regulate multiple target genes, and a powerful inflammation-related lncRNA might also target multiple inflammation genes, for example, H19 targets TGFβ1 in prostate cancer cells [55]; TGFβ1 is a key regulator in CCA inflammation [1], suggesting the pivotal role of inflammation regulation by H19. Therefore, we speculate that H19 and HULC may act as primary regulators of other downstream inflammation genes that initiate and sustain CCA.

The growing evidences have indicated that H19 involved in both proliferation and differentiation processes, together with epithelial to mesenchymal transition (EMT) and also mesenchymal to epithelial transition (MET), suggesting its contribution in tumor both initiation and progression [56]. H19 has an evolutionary conservation secondary structure, suggesting its structure-dependent functions, which include binding to enhancer of zeste homolog 2 (EZH2) [57], and interacting with the methyl-CpG-binding domain protein 1 (MBD1) and recruiting it to some of its targets that maintain the repressive H3K9me3 histone marks in their loci [58]. H19 has also been recently found to interact with P53 proteins, and led to its inactivation, indicating that H19 plays a role in tumorigenesis [59]. HULC was reported to promote the proliferation and regulated cell cycle of HCC through down-regulation of the tumor suppressor gene CDKN2C (p18) and involved in signaling pathways including ATM/ATR and p53 [60, 61]. In this study, we revealed the potential pathway of H19 that is targeted by let-7a/let-7b, causing the partial inactivation of IL-6 in its mediated inflammatory responses triggered by oxidative stress in cholangiocarcinoma. We also found that HULC was targeted by miR-372 and miR-373 and further activated the chemokine receptor CXCR4 in the progression of migration and invasion in cholangiocarcinoma cells. The results suggest a potential pathway of lncRNAs participated in the progression of CCA.

Previous studies indicated that oxidative stress from infection with parasites, bacteria and viruses is highly related to the inflammatory and malignant processes of cholangiocytes [7, 62, 63], and cholangiocytes under oxidative stress might induce and augment inflammatory responses [7, 62]. An increasing number of studies have realized that inflammation is one of important responses to the oxidative stress in many diseases, such as the gut [64], lung [65], pancreatitis [66], colorectal cancer [49], and cholangiocarcinoma [67]. Fernanda et al. also reviewed a total of 1332 studies initially identified that TNF-a, IL-8, IL-6, IL-1b, and NF-κB were the main inflammatory mediators and oxidative stress markers [65], suggesting that the oxidative stress made a great contribution in inflammatory in the initiation or progression of disease. However, the molecular details between oxidative stress and inflammation are not well defined in CCA cells. Several previous studies have indicated that infections are induced by oxidative stress via chronic inflammation [5, 7]. In this study, we found that H19 and HULC are stimulated by both short-term and long-term oxidative stress and regulate the expression of pivotal genes in the inflammatory process, suggesting that there is a positive feedback loop between inflammation and oxidative stress, and the activation of this feedback loop with lncRNAs might promote tumorigenesis in CCA.

## Conclusions

Our study revealed that H19 and HULC were up-regulated by oxidative stress and target IL-6 and CXCR4 via ceRNA patterns of sponging let-7a/let-7b and miR-372/miR-373, respectively. Furthermore, overexpressed H19 and HULC might promote cell migration and invasion, suggesting that these lncRNAs might be the chief culprits of CCA pathogenesis and progression (Fig. 5). Our results provide new insight into the mechanism linking lncRNA function with CCA and may serve as novel targets for the development of new countermeasures of CCA.

## Additional file

Additional file 1: Figure S1. The expression of heme oxygenase-1 (HO-1) had increased significantly under the oxidative stress condition. HO-1 stimulated by short-term oxidative stress using H2O2 (A) and long-term oxidative stress using glucose oxidase (B) in QBC939, SK-cha-1, and RBE cells. Figure S2. qPCR validations of forced expressed H19 and HULC in CCA cells (A) and knockdown of lncRNAs, H19, and HULC in RBE cells by SiRNAs. Figure S3. Knockdown of H19 and HULC expression decreased the ability of migration and invasion in CCA cells. Migration assays of CCA cells (A). Invasion assays of CCA cells (B). Figure S4. MiRNAs targeted inflammation-associated genes, IL-6 and CXCR4. A. Schematic representation of the interaction between miRNAs and H19 or HULC (A). Let-7a/let-7b and miR-372/miR-373 suppressed luciferase activity of vectors with 3'-UTR of IL-6 and CXCR4 in RBE cells (B). Figure S5. The histogram of miRNA down-regulation by transfecting the miRNA inhibitors into RBE cells under oxidative stress. The expression levels of IL-6 (A) and CXCR4 (B) increased significantly. Figure S6. Western Blot validations of forced expressed IL-6 and CXCR4 in CCA cells. CXCR4-1 and CXCR4-2 represent two alternative splicing of CXCR4. The right histogram of relative gray values represented the protein level. Figure S7. Enforced IL-6 and CXCR4 expression increased the ability of invasion in CCA cells. Scratch wound healing assays of CCA cells (A). Migration assays of CCA cells (B). Invasion assays of CCA cells (C). Table S1. Primer sequence for qPCR and vector construction. (PDF 1109 kb)

## Abbreviations

CCA: Cholangiocarcinoma
ceRNAs: Competing endogenous RNAs
EMT: Epithelial to mesenchymal transition
EZH2: Enhancer of zeste homolog 2
H3K27: Histone H3 Lys 27
HBV: Hepatitis B virus
HO-1: heme oxygenase-1
lncRNAs: Long non-coding RNAs
MBD1: Methyl-CpG-binding domain protein 1
MET: Mesenchymal to epithelial transition
miRNAs: microRNAs
NF-κB: Nuclear factor kappa B
RIP: RNA immunoprecipitation
ROS: Reactive oxygen species
STAT3: Signal transducer and activator of transcription 3

## References

[1] Fava G, Lorenzini I (2012). Molecular pathogenesis of cholangiocarcinoma. Int J Hepatol.

[2] Karin M, Clevers H (2016). Reparative inflammation takes charge of tissue regeneration. Nature..

[3] Pinlaor S, Ma N, Hiraku Y, Yongvanit P, Semba R, Oikawa S (2004). Repeated infection with Opisthorchis viverrini induces accumulation of 8-nitroguanine and 8-oxo-7,8-dihydro-2′-deoxyguanine in the bile duct of hamsters via inducible nitric oxide synthase. Carcinogenesis.

[4] Rizvi S, Gores GJ (2013). Pathogenesis, diagnosis, and management of cholangiocarcinoma. Gastroenterology.

[5] Kawanishi S, Hiraku Y, Pinlaor S, Ma N (2006). Oxidative and nitrative DNA damage in animals and patients with inflammatory diseases in relation to inflammation-related carcinogenesis. Biol Chem.

[6] Pinlaor S, Hiraku Y, Yongvanit P, Tada-Oikawa S, Ma N, Pinlaor P (2006). iNOS-dependent DNA damage via NF-kappaB expression in hamsters infected with Opisthorchis viverrini and its suppression by the antihelminthic drug praziquantel. Int J Cancer.

[7] Reuter S, Gupta SC, Chaturvedi MM, Aggarwal BB (2010). Oxidative stress, inflammation, and cancer: how are they linked?. Free Radic Biol Med.

[8] Yongvanit P, Pinlaor S, Bartsch H (2012). Oxidative and nitrative DNA damage: key events in opisthorchiasis-induced carcinogenesis. Parasitol Int.

[9] Tarocchi M, Polvani S, Marroncini G, Galli A (2014). Molecular mechanism of hepatitis B virus-induced hepatocarcinogenesis. World J Gastroenterol.

[10] Cui H, Xie N, Tan Z, Banerjee S, Thannickal VJ, Abraham E (2014). The human long noncoding RNA lnc-IL7R regulates the inflammatory response. Eur J Immunol.

[11] Krawczyk M, Emerson BM (2014). p50-associated COX-2 extragenic RNA (PACER) activates COX-2 gene expression by occluding repressive NF-kappaB complexes. Elife.

[12] Liu Y, Luo F, Xu Y, Wang B, Zhao Y, Xu W (2015). Epithelial-mesenchymal transition and cancer stem cells, mediated by a long non-coding RNA, HOTAIR, are involved in cell malignant transformation induced by cigarette smoke extract. Toxicol Appl Pharmacol.

[13] Manca S, Magrelli A, Cialfi S, Lefort K, Ambra R, Alimandi M (2011). Oxidative stress activation of miR-125b is part of the molecular switch for Hailey-Hailey disease manifestation. Exp Dermatol.

[14] Yarmishyn AA, Batagov AO, Tan JZ, Sundaram GM, Sampath P, Kuznetsov VA (2014). HOXD-AS1 is a novel lncRNA encoded in HOXD cluster and a marker of neuroblastoma progression revealed via integrative analysis of noncoding transcriptome. BMC Genomics.

[15] Chen L, Yan HX, Yang W, Hu L, Yu LX, Liu Q (2009). The role of microRNA expression pattern in human intrahepatic cholangiocarcinoma. J Hepatol.

[16] Mishra PJ (2014). MicroRNAs as promising biomarkers in cancer diagnostics. Biomark Res.

[17] Meng F, Wehbe-Janek H, Henson R, Smith H, Patel T (2008). Epigenetic regulation of microRNA-370 by interleukin-6 in malignant human cholangiocytes. Oncogene.

[18] Chen Y, Luo J, Tian R, Sun H, Zou S (2011). miR-373 negatively regulates methyl-CpG-binding domain protein 2 (MBD2) in hilar cholangiocarcinoma. Dig Dis Sci.

[19] Chen YJ, Luo J, Yang GY, Yang K, Wen SQ, Zou SQ (2012). Mutual regulation between microRNA-373 and methyl-CpG-binding domain protein 2 in hilar cholangiocarcinoma. World J Gastroenterol.

[20] Chusorn P, Namwat N, Loilome W, Techasen A, Pairojkul C, Khuntikeo N (2013). Overexpression of microRNA-21 regulating PDCD4 during tumorigenesis of liver fluke-associated cholangiocarcinoma contributes to tumor growth and metastasis. Tumour Biol.

[21] Namwat N, Chusorn P, Loilome W, Techasen A, Puetkasichonpasutha J, Pairojkul C (2012). Expression profiles of oncomir miR-21 and tumor suppressor let-7a in the progression of opisthorchiasis-associated cholangiocarcinoma. Asian Pac J Cancer Prev.

[22] Huang MD, Chen WM, Qi FZ, Xia R, Sun M, Xu TP (2015). Long non-coding RNA ANRIL is upregulated in hepatocellular carcinoma and regulates cell apoptosis by epigenetic silencing of KLF2. J Hematol Oncol.

[23] Loewen G, Jayawickramarajah J, Zhuo Y, Shan B (2014). Functions of lncRNA HOTAIR in lung cancer. J Hematol Oncol.

[24] Iizuka N, Oka M, Tamesa T, Hamamoto Y, Yamada-Okabe H (2004). Imbalance in expression levels of insulin-like growth factor 2 and H19 transcripts linked to progression of hepatocellular carcinoma. Anticancer Res.

[25] Han BW, Chen YQ (2013). Potential pathological and functional links between long noncoding RNAs and hematopoiesis. Sci Signal.

[26] Yang F, Zhang L, Huo XS, Yuan JH, Xu D, Yuan SX (2011). Long noncoding RNA high expression in hepatocellular carcinoma facilitates tumor growth through enhancer of zeste homolog 2 in humans. Hepatology.

[27] Wang J, Liu X, Wu H, Ni P, Gu Z, Qiao Y (2010). CREB up-regulates long non-coding RNA, HULC expression through interaction with microRNA-372 in liver cancer. Nucleic Acids Res.

[28] Li G, Zhang H, Wan X, Yang X, Zhu C, Wang A (2014). Long noncoding RNA plays a key role in metastasis and prognosis of hepatocellular carcinoma. Biomed Res Int.

[29] Voellenkle C, Garcia-Manteiga JM, Pedrotti S, Perfetti A, De Toma I, Da SD (2016). Implication of Long noncoding RNAs in the endothelial cell response to hypoxia revealed by RNA-sequencing. Sci Rep.

[30] Liu B, Sun L, Liu Q, Gong C, Yao Y, Lv X (2015). A cytoplasmic NF-kappaB interacting long noncoding RNA blocks IkappaB phosphorylation and suppresses breast cancer metastasis. Cancer Cell.

[31] Mirza AH, Berthelsen CH, Seemann SE, Pan X, Frederiksen KS, Vilien M (2015). Transcriptomic landscape of lncRNAs in inflammatory bowel disease. Genome Med.

[32] McKiernan PJ, Molloy K, Cryan SA, McElvaney NG, Greene CM (2014). Long noncoding RNA are aberrantly expressed in vivo in the cystic fibrosis bronchial epithelium. Int J Biochem Cell Biol.

[33] Lin J, Zhang X, Xue C, Zhang H, Shashaty MG, Gosai SJ (2015). The long noncoding RNA landscape in hypoxic and inflammatory renal epithelial injury. Am J Physiol Renal Physiol.

[34] de Oliveira-Marques V, Cyrne L, Marinho HS, Antunes F (2007). A quantitative study of NF-kappaB activation by H2O2: relevance in inflammation and synergy with TNF-alpha. J Immunol.

[35] Minamino T, Christou H, Hsieh CM, Liu Y, Dhawan V, Abraham NG (2001). Targeted expression of heme oxygenase-1 prevents the pulmonary inflammatory and vascular responses to hypoxia. Proc Natl Acad Sci U S A.

[36] Kongpetch S, Puapairoj A, Ong CK, Senggunprai L, Prawan A, Kukongviriyapan U (2016). Haem oxygenase 1 expression is associated with prognosis in cholangiocarcinoma patients and with drug sensitivity in xenografted mice. Cell Prolif.

[37] Uchida D, Takaki A, Ishikawa H, Tomono Y, Kato H, Tsutsumi K (2016). Oxidative stress balance is dysregulated and represents an additional target for treating cholangiocarcinoma. Free Radic Res.

[38] Sanchez-Mejias A, Tay Y (2015). Competing endogenous RNA networks: tying the essential knots for cancer biology and therapeutics. J Hematol Oncol.

[39] Kallen AN, Zhou XB, Xu J, Qiao C, Ma J, Yan L (2013). The imprinted H19 lncRNA antagonizes let-7 microRNAs. Mol Cell.

[40] Johnson C, Han Y, Hughart N, McCarra J, Alpini G, Meng F (2012). Interleukin-6 and its receptor, key players in hepatobiliary inflammation and cancer. Transl Gastrointest Cancer.

[41] Zhao S, Wang J, Qin C (2014). Blockade of CXCL12/CXCR4 signaling inhibits intrahepatic cholangiocarcinoma progression and metastasis via inactivation of canonical Wnt pathway. J Exp Clin Cancer Res.

[42] Wang P, Xue Y, Han Y, Lin L, Wu C, Xu S (2014). The STAT3-binding long noncoding RNA lnc-DC controls human dendritic cell differentiation. Science.

[43] Fang K, Han BW, Chen ZH, Lin KY, Zeng CW, Li XJ (2014). A distinct set of long non-coding RNAs in childhood MLL-rearranged acute lymphoblastic leukemia: biology and epigenetic target. Hum Mol Genet.

[44] Xu TP, Huang MD, Xia R, Liu XX, Sun M, Yin L (2014). Decreased expression of the long non-coding RNA FENDRR is associated with poor prognosis in gastric cancer and FENDRR regulates gastric cancer cell metastasis by affecting fibronectin 1 expression. J Hematol Oncol.

[45] Fan Y, Wang YF, Su HF, Fang N, Zou C, Li WF (2016). Decreased expression of the long noncoding RNA LINC00261 indicate poor prognosis in gastric cancer and suppress gastric cancer metastasis by affecting the epithelial-mesenchymal transition. J Hematol Oncol.

[46] Eichten A, Su J, Adler AP, Zhang L, Ioffe E, Parveen AA (2016). Resistance to anti-VEGF therapy mediated by autocrine IL6/STAT3 signaling and overcome by IL6 blockade. Cancer Res.

[47] Landskron G, De la Fuente M, Thuwajit P, Thuwajit C, Hermoso MA (2014). Chronic inflammation and cytokines in the tumor microenvironment. J Immunol Res.

[48] Kim SY, Kang JW, Song X, Kim BK, Yoo YD, Kwon YT (2013). Role of the IL-6-JAK1-STAT3-Oct-4 pathway in the conversion of non-stem cancer cells into cancer stem-like cells. Cell Signal.

[49] Lin KY, Ye H, Han BW, Wang WT, Wei PP, He B (2016). Genome-wide screen identified let-7c/miR-99a/miR-125b regulating tumor progression and stem-like properties in cholangiocarcinoma. Oncogene.

[50] Goydos JS, Brumfield AM, Frezza E, Booth A, Lotze MT, Carty SE (1998). Marked elevation of serum interleukin-6 in patients with cholangiocarcinoma: validation of utility as a clinical marker. Ann Surg.

[51] Rosen HR, Winkle PJ, Kendall BJ, Diehl DL (1997). Biliary interleukin-6 and tumor necrosis factor-alpha in patients undergoing endoscopic retrograde cholangiopancreatography. Dig Dis Sci.

[52] Hummel S, Van Aken H, Zarbock A (2014). Inhibitors of CXC chemokine receptor type 4: putative therapeutic approaches in inflammatory diseases. Curr Opin Hematol.

[53] Leelawat K, Leelawat S, Narong S, Hongeng S (2007). Roles of the MEK1/2 and AKT pathways in CXCL12/CXCR4 induced cholangiocarcinoma cell invasion. World J Gastroenterol.

[54] Ohira S, Sasaki M, Harada K, Sato Y, Zen Y, Isse K (2006). Possible regulation of migration of intrahepatic cholangiocarcinoma cells by interaction of CXCR4 expressed in carcinoma cells with tumor necrosis factor-alpha and stromal-derived factor-1 released in stroma. Am J Pathol.

[55] Matouk IJ, Raveh E, Abu-lail R, Mezan S, Gilon M, Gershtain E (1843). Oncofetal H19 RNA promotes tumor metastasis. Biochim Biophys Acta.

[56] Raveh E, Matouk IJ, Gilon M, Hochberg A (2015). The H19 Long non-coding RNA in cancer initiation, progression and metastasis—a proposed unifying theory. Mol Cancer.

[57] Luo M, Li Z, Wang W, Zeng Y, Liu Z, Qiu J (2013). Long non-coding RNA H19 increases bladder cancer metastasis by associating with EZH2 and inhibiting E-cadherin expression. Cancer Lett.

[58] Monnier P, Martinet C, Pontis J, Stancheva I, Ait-Si-Ali S, Dandolo L (2013). H19 lncRNA controls gene expression of the Imprinted Gene Network by recruiting MBD1. Proc Natl Acad Sci U S A.

[59] Yang F, Bi J, Xue X, Zheng L, Zhi K, Hua J (2012). Up-regulated long non-coding RNA H19 contributes to proliferation of gastric cancer cells. FEBS J.

[60] Du Y, Kong G, You X, Zhang S, Zhang T, Gao Y (2012). Elevation of highly up-regulated in liver cancer (HULC) by hepatitis B virus X protein promotes hepatoma cell proliferation via down-regulating p18. J Biol Chem.

[61] Li C, Chen J, Zhang K, Feng B, Wang R, Chen L (2015). Progress and prospects of long noncoding RNAs (lncRNAs) in hepatocellular carcinoma. Cell Physiol Biochem.

[62] Di Virgilio F (2004). New pathways for reactive oxygen species generation in inflammation and potential novel pharmacological targets. Curr Pharm Des.

[63] Thanan R, Pairojkul C, Pinlaor S, Khuntikeo N, Wongkham C, Sripa B (2013). Inflammation-related DNA damage and expression of CD133 and Oct3/4 in cholangiocarcinoma patients with poor prognosis. Free Radic Biol Med.

[64] Barrett CW, Short SP, Williams CS. Selenoproteins and oxidative stress-induced inflammatory tumorigenesis in the gut. Cell Mol Life Sci. 2016. [Epub ahead of print]. 10.1007/s00018-016-2339-2 PMC5274549 27563706

[65] de Carvalho FO, Felipe FA, de Melo Costa ACS, Teixeira LGB, Silva ÉR, Nunes PS (2016). Inflammatory mediators and oxidative stress in animals subjected to smoke inhalation: a systematic review. Lung.

[66] Yu JH (2014). Oxidative stress and inflammatory signaling in cerulein pancreatitis. World J Gastroentero.

[67] Prawan A, Buranrat B, Kukongviriyapan U, Sripa B, Kukongviriyapan V (2009). Inflammatory cytokines suppress NAD(P)H:quinone oxidoreductase-1 and induce oxidative stress in cholangiocarcinoma cells. J Cancer Res Clin Oncol.

