# Supplementary material for: LncRNAs H19 and HULC, activated by oxidative stress, promote cell migration and invasion in cholangiocarcinoma through a ceRNA manner
Source: J Hematol Oncol. 2016 Nov 3;9:117. doi: 10.1186/s13045-016-0348-0 (PMC5093965; doi:10.1186/s13045-016-0348-0)
Supplement: Additional file 1: Figure S1. — The expression of heme oxygenase-1 (HO-1) had increased significantly under the oxidative stress condition. HO-1 stimulated by short-term oxidative stress using H2O2 (A) and long-term oxidative stress using glucose oxidase (B) in QBC939, SK-cha-1, and RBE cells. Figure S2. qPCR validations of forced expressed H19 and HULC in CCA cells (A) and knockdown of lncRNAs, H19, and HULC in RBE cells by SiRNAs. Figure S3. Knockdown of H19 and HULC expression decreased the ability of migration and invasion in CCA cells. Migration assays of CCA cells (A). Invasion assays of CCA cells (B). Figure S4. MiRNAs targeted inflammation-associated genes, IL-6 and CXCR4. A. Schematic representation of the interaction between miRNAs and H19 or HULC (A). Let-7a/let-7b and miR-372/miR-373 suppressed luciferase activity of vectors with 3'-UTR of IL-6 and CXCR4 in RBE cells (B). Figure S5. The histogram of miRNA down-regulation by transfecting the miRNA inhibitors into RBE cells under oxidative stress. The expression levels of IL-6 (A) and CXCR4 (B) increased significantly. Figure S6. Western Blot validations of forced expressed IL-6 and CXCR4 in CCA cells. CXCR4-1 and CXCR4-2 represent two alternative splicing of CXCR4. The right histogram of relative gray values represented the protein level. Figure S7. Enforced IL-6 and CXCR4 expression increased the ability of invasion in CCA cells. Scratch wound healing assays of CCA cells (A). Migration assays of CCA cells (B). Invasion assays of CCA cells (C). Table S1. Primer sequence for qPCR and vector construction. (PDF 1109 kb) [file 13045_2016_348_MOESM1_ESM.pdf]

LncRNAs H19 and HULC, activated by oxidative stress, promote cell migration and invasion in cholangiocarcinoma through a ceRNA manner

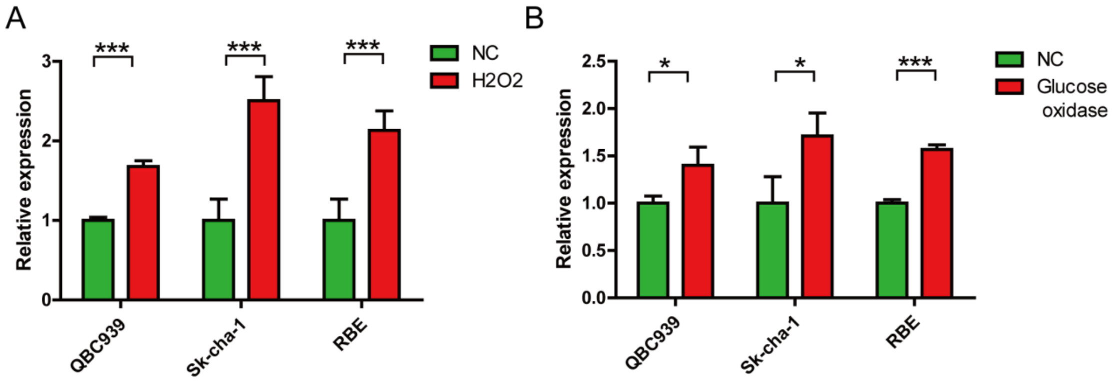

Figure S1. The expression of heme oxygenase-1(HO-1) had increased significantly under the oxidative stress condition. HO-1 stimulated by short-term oxidative stress using H_2O_2 (A) and long-term oxidative stress using glucose oxidase (B) in QBC939, SK-cha-1 and RBE cells.

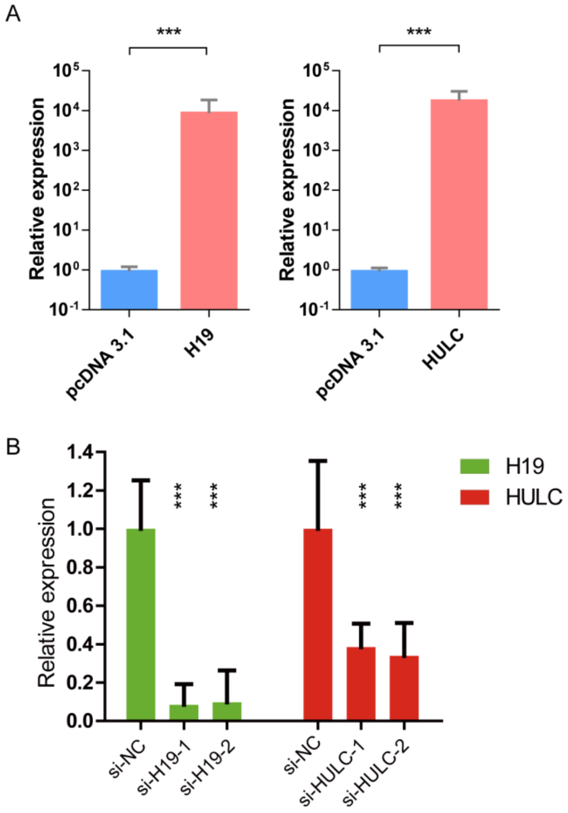

Figure S2. qPCR validations of forced expressed H19 and HULC in CCA cells（A）, and knockdown of lncRNAs, H19 and HULC in RBE cells by SiRNAs.

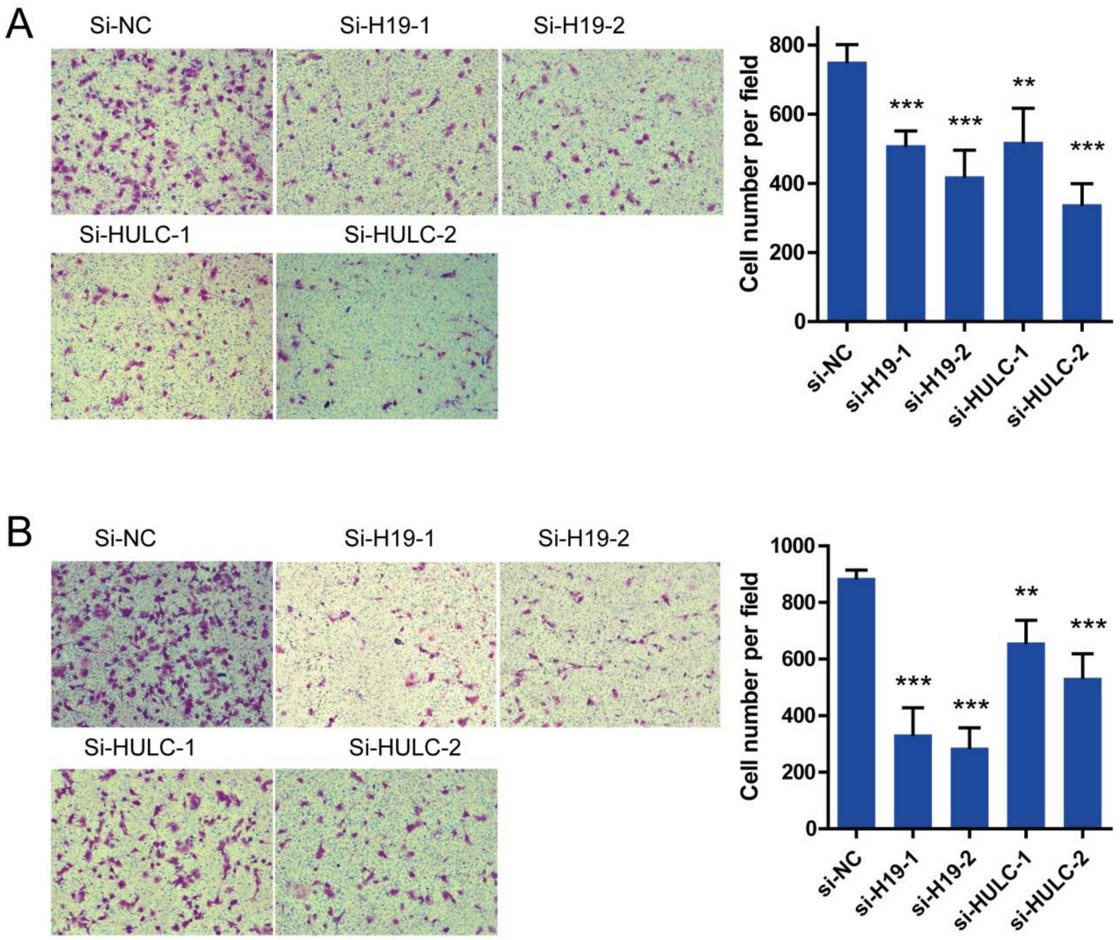

Figure S3. Knockdown of H19 and HULC expression decreased the ability of migration and invasion in CCA cells.

A. Migration assays of CCA cells. B. Invasion assays of CCA cells.

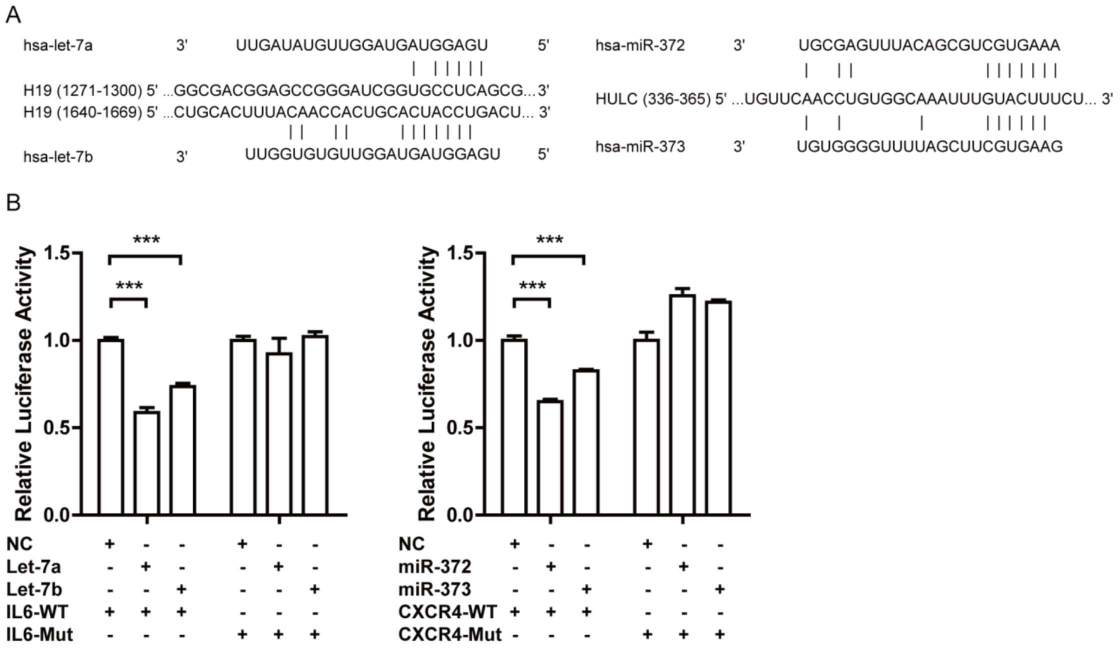

Figure S4. **MiRNAs targeted Inflammation associated genes, IL6 and CXCR4.** A. Schematic representation of the interaction between miRNAs and H19 or HULC. B. Let-7a/let-7b and miR-372/miR-373 suppressed luciferase activity of vectors with 3' UTR of IL6 and CXCR4 in RBE cells.

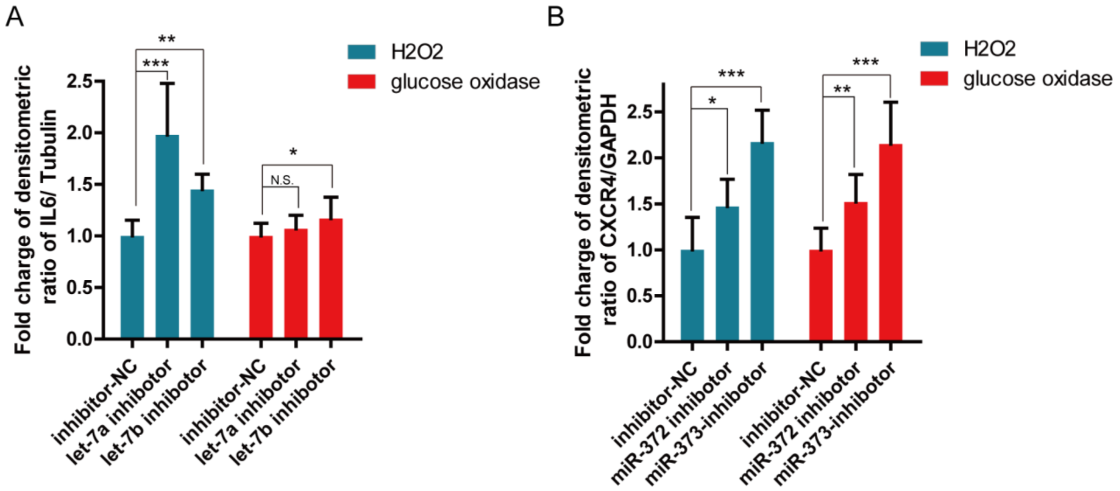

Figure S5. The histogram of miRNA downregulation by transfecting the miRNA inhibitors into RBE cells under oxidative stress. The expression levels of IL6 (A) and CXCR4 (B) increased significantly.

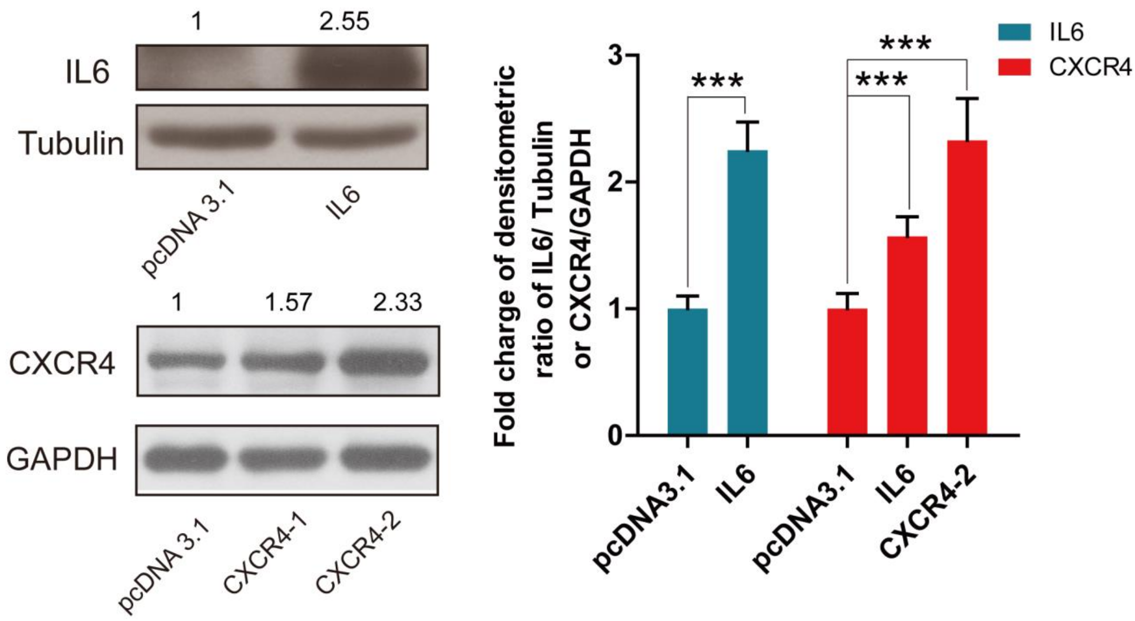

Figure S6. Western Blot validations of forced expressed IL6 and CXCR4 in CCA cells. CXCR4-1 and CXCR4-2 represent two alternative splicing of CXCR4. The right histogram of relative gray values represented the protein level.

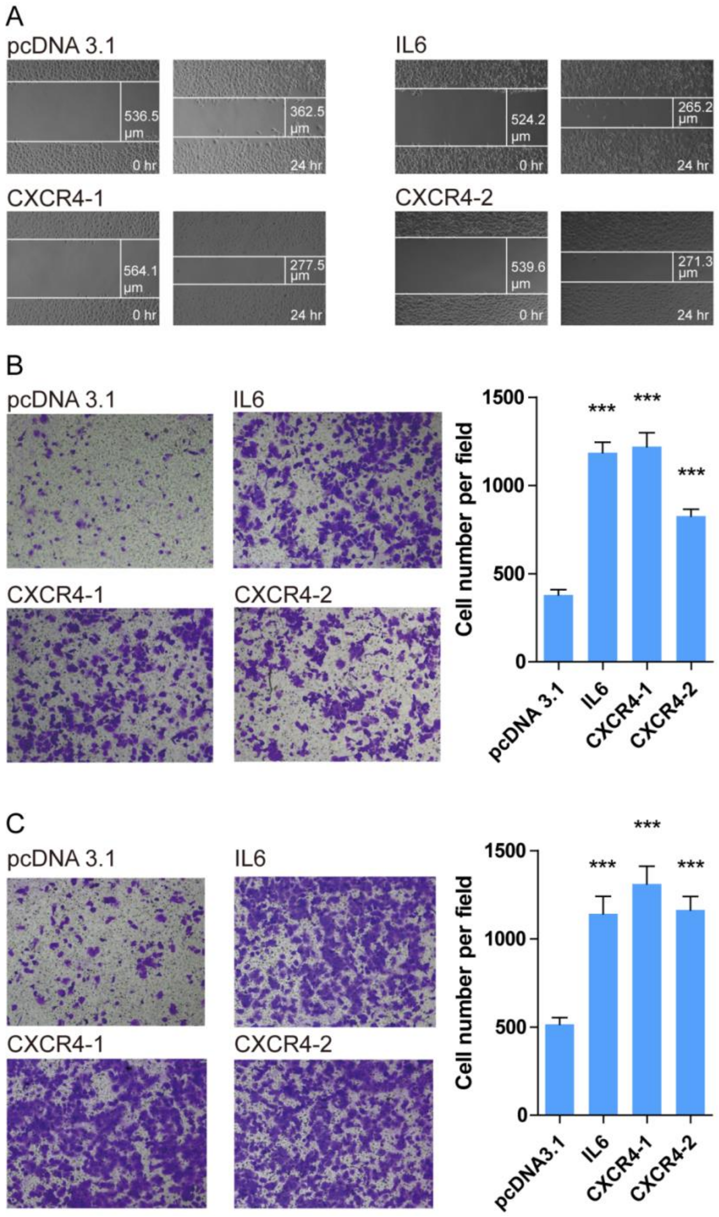

Figure S7. Enforced IL6 and CXCR4 expression increased the ability of invasion in CCA cells. A Scratch wound healing assays of CCA cells. B Migration assays of CCA cells. C Invasion assays of CCA cells.

Table S1. Primer sequence for qPCR and vector construction.

Primer Name	Sequence 5' to 3'
**Primers for qPCR**	
qHULC-Forward	GCAAGCCAGGAAGAGTCGTC
qHULC-Reverse	GCTGTGCTTAGTTTATTGCCAGG
qH19-Forward	TTCCAGGCAGAAAGAGCAAGAGGGC
qH19-Reverse	AGACGTCCTGCTGCAACTCCCCGAG
qIL6-Forward	GGTACATCCTCGACGGCATCTC
qIL6-Reverse	CTCTGGCTTGTTCCTCACTACT

qCXCR4-Forward	CACGCCACCAACAGTCAGA
qCXCR4-Reverse	ACAATACCAGGCAGGATAAGG
qGAPDH-Forward	GCTGAACGGGAAGCTCACTG
qGAPDH-Reverse	GTGCTCAGTGTAGCCCAGGA
qHO-1- Forward	CAACATTGAGCTGTTTGAGG
qHO-1- Reverse	TGGTCTTTGTGTTCCTCTGTC
**Primers for full-length amplification**	
HULC-Forward	AGTCGAATTCATGGGGGTGGAACTCATGATGGAAT
HULC-Reverse	GATCCTCGAGTTAAGAATGGACATCATTTTATTTC
IL6-Forward	CGCGGATCCAGGACTGGAGATGTCTGAGGCT
IL6-Reverse	CCGGAATTCTGATCTGGCTCTGAAACAAAGG
CXCR4-1-Forward	AGTCGAATTCATGGAGGGGATCAGTATA
CXCR4-2-Forward	AGTCGAATTCATGTCCATTCCTTTGCCT
CXCR4-Reverse	GATCCTCGAGTTAGCTGGAGTGAAAACTTG
**Oligonucleotides for 3' UTR construction**	
CXCR4-miR372373-WT-Sense	TCGAGCTTAAGACGTGATTTTGCTGTAGAAGATGGCACTTATAACCAAAGCCCAAAGGC
CXCR4-miR372373-WT-Antisense	GGCCGCCTTTGGGCTTTGGTTATAAGTGCCATCTTCTACAGCAAAATCACGTCTTAAGC
CXCR4-miR372373-Mut-Sense	TCGAGCTTAAGACGTGATTTTGCTGTAGAAGATGGTATTCATAACCAAAGCCCAAAGGC
CXCR4-miR372373-Mut-Antisense	GGCCGCCTTTGGGCTTTGGTTATGAATACCATCTTCTACAGCAAAATCACGTCTTAAGC
IL6-let7ab-WT-Sense	TCGAGCCAGATCATTTCTTGGAAAGTGTAGGCTTACCTCAAATAAATGGCTAACTTAGC
IL6-let7ab-WT-Antisense	GGCCGCTAAGTTAGCCATTTATTTGAGGTAAGCCTACACTTTCCAAGAAATGATCTGGC
IL6-let7ab-Mut-Sense	TCGAGCCAGATCATTTCTTGGAAAGTGTAGGCTTTCGTGAAATAAATGGCTAACTTAGC
IL6-let7ab-Mut-Antisense	GGCCGCTAAGTTAGCCATTTATTTCACGAAAGCCTACACTTTCCAAGAAATGATCTGGC
